# Meteorological Register for July, 1817

**Published:** 1817-10

**Authors:** John Bailey

**Affiliations:** Surgeon, &c.


### Meteorological Register for July, 1817.

*Kept at Harwich, in Essex.*

By JOHN BAILEY, Surgeon, &c.

| | Moon's Age. | Atmospherical Pressure. Morn. | Ev. | Temp. | Wind. | Remarks. | General Statement of Diseases. |
|---|---|---|---|---|---|---|---|
| | 1 | 29 7 | 29 5 | 63 | SW | Rain | Rheumatism. |
| | 2 | 5 | 8 | 66 | W | Gale of wind | Acutus |
| | 3 | 8 | 6½ | 68 | W | Showery | Cholera |
| | 4 | 6 | 6 | 70 | W | Fine | |
| | 5 | 5 | 6 | 65 | NW | Gentle showers | |
| ☽ | 6 | 6½ | 6½ | 64 | W | ditto | |
| | 7 | 6½ | 6½ | 70 | W | Fine & clear | |
| | 8 | 7 | 8 | 69 | W | | |
| | 9 | 8 | 8 | 67 | W | Cloudy | |
| | 10 | | | 70 | W | Fine & clear | |
| | 11 | 7 | 7 | 73 | W | Showery | |
| | 12 | 7 | 9 | 65 | E | Rain | |
| | 13 | 9 | 6 | 71 | NE | ditto | |
| ● | 14 | 6 | 5 | 66 | S | ditto | |
| | 15 | 3 | 2 | 65 | W | Much rain & W. | |
| | 16 | 4½ | 7 | 61 | NW | Cloudy, with R. | |
| | 17 | 7 | 7 | 67 | W | ditto | |
| | 18 | 7 | 8 | 67 | N | Violent shower | |
| | 19 | 8½ | 8½ | 64 | N | Rain | |
| | 20 | 9 | 9 | 69 | NW | ditto | |
| ☾ | 21 | 8½ | 8½ | 68 | W | ditto | |
| | 22 | 7½ | 7½ | 68 | SW | ditto | |
| | 23 | 8 | 9 | 67 | W | ditto | |
| | 24 | 9 | 9½ | 67 | W | ditto | |
| | 25 | 9½ | 9½ | 70 | W | ditto | |
| | 26 | 8½ | 6 | 63 | SW | ditto | |
| | 27 | 6 | 6 | 65 | SW | ditto | |
| ○ | 28 | 7½ | 8½ | 66 | SW | Stormy, with R. | |
| | 29 | 9 | 9 | 67 | SW | Rain | |
| | 30 | 7 | 7 | 68 | SW | ditto | |
| | 31 | | | 67 | SW | ditto | |

The list of diseases for this month is less varied than usual: owing, it is presumed, to the chilliness which a long descent of rain and its evaporation produce, the acute form of rheumatism has been a common and an obstinate ailment. This disorder rarely visits us in the Estival quarter of the year; the wet and muggy weather of a mild winter, alternating with keen north-west and easterly winds, brings its usual harbingers.—Cholera, which succeeds to summer heats, and is the autumnal endemic of this flat and marshy county, has paid its visit sooner than usual; a great many cases have occurred, wearing all the different shades of character from simple tormina to the more aggravated form of dysenteria.

